# Continuous-Flow Solar Heterogeneous Photo-Fenton Process in a Lab-Made Low-Cost CPC Photoreactor for Efficient Dye Degradation

**DOI:** 10.1021/acsomega.5c03994

**Published:** 2025-07-15

**Authors:** Matheus Gabriel Guardiano, Yara Silvestrine e Silva, Rossano Gimenes, Sandro José de Andrade, Márcia Matiko Kondo, Milady Renata Apolinário da Silva

**Affiliations:** 425908 Federal University of Itajubá, Av. BPS, 1303, Itajubá, MG 37500-901, Brazil

## Abstract

In this work, a continuous-flow lab-made solar compound parabolic concentrator (CPC) photoreactor was designed to be used in a heterogeneous solar photo-Fenton process. The photoreactor was constructed by using recyclable and low-cost materials. Mn-Zn ferrite obtained from spent batteries was used as the catalyst, and H_2_O_2_ was used as the oxidizing agent to evaluate the CPC photoreactor using methylene blue (MB) as the target compound. The ferrite was securely attached to the central position of the tubes using neodymium magnetic bars, while an aquarium pump was employed to propel the fluid through the tubes, ensuring adequate velocity for the photo-Fenton treatment. The results demonstrated some important parameters that needed to be considered during CPC photoreactor construction, such as reflective surface dimensions and concentration factor. Parameters such as total organic carbon, discoloration, and chemical oxygen demand were evaluated in order to assess the MB removal efficiency. The results showed that after 1 h of solar irradiation, 98% of MB was degraded. The catalyst composition before and after the application process was investigated by using X-ray photoelectron spectroscopy analysis, revealing that the enhanced degradation was associated with the participation of Fe, Mn, and Zn in the ferrite performance for hydroxyl radical generation. The photoreactor demonstrated good versatility and applicability, making it suitable for wastewater treatment and investigation of various photocatalytic processes that used solar irradiation.

## Graphical abstract

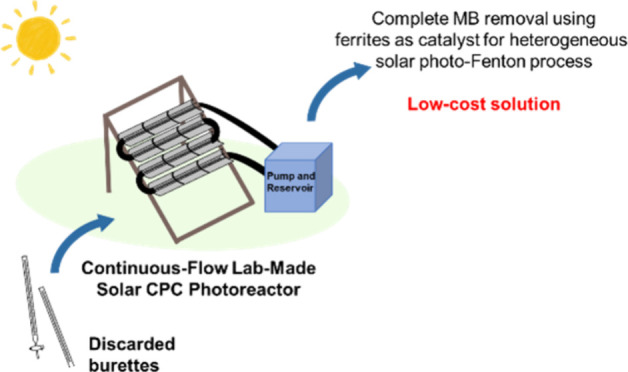

## Introduction

The textile industry has high consumption of water and can generate a significant amount of effluent during its production process.^1^ The volume of effluent produced by industries is a significant concern due to its high concentration of organic chemical compounds and a variety of complex contaminants. These substances exhibit high chemical resistance and persistence, making the biodegradation processes, therefore, more difficult.^2^ Dyes, in general, are the main components of these effluents, and they are known to be challenging to degrade and highly toxic to the environment.^3,4^

The disposal of untreated effluents containing those dyes in aquatic environments can rapidly disrupt ecosystem balance. The presence of dyes in water hinders the penetration of sunlight into deeper layers, thereby altering the photosynthetic activity of the environment.^5^ Consequently, this effect can lead to a deterioration in water quality and an increase in toxic effects on aquatic fauna and flora.^6^ Conventional wastewater treatment processes (physical-chemical and/or biological) are often applied to dye-containing effluents; however, they exhibit low efficiency in color removal.^7^ Therefore, an alternative treatment such as advanced oxidation processes (AOPs) or adsorption processes have been studied when trying to remove dyes from effluents after the conventional treatment.^8^ The AOPs can oxidize the organics to smaller compounds or even mineralize them to water, carbon dioxide, and inorganic anions.^9^ The generated smaller compounds may include transformation products, whether toxic or not, as well as carboxylic acids that recently have gained attention in biomass valorization studies.^10–12^ As for the adsorption processes, they do not destroy the pollutant—they only transfer it from the aqueous phase to the solid one.^13,14^

Among the AOPs, the Fenton and photo-Fenton processes are efficiently used to remove various compounds, such as chlorinated aliphatics, chlorinated aromatics, polychlorinated biphenyls, nitroaromatics, azo dyes, chlorobenzene, and phenols.^15^ These processes can achieve complete degradation of the dyes and partial degradation of the organic matter within a reduced reaction time.^16^ The Fenton reaction involves the catalytic oxidation of organic compounds in the presence of hydrogen peroxide and ferrous salts (eq 1).^17,18^ The efficiency of organic compound degradation through Fenton reactions can be enhanced by utilizing a source of UV or solar radiation, known as the photo-Fenton process (eq 2).^9^ Additionally, the immobilization of iron or the use of iron-containing other materials as catalysts leads to the process known as the heterogeneous Fenton reaction, which overcomes some disadvantages of the homogeneous process, such as the sludge generation after treatment.^19^

$${\mathrm{Fe}}^{2+}+H_{2}O_{2}\to {\mathrm{Fe}}^{3+}+{\mathrm{HO}}^{\cdot }+{\mathrm{HO}}^{-}$$ 1

$$\mathrm{Fe}(\mathrm{OH}{)}^{2+}+h\nu \to {\mathrm{Fe}}^{2+}+{\mathrm{HO}}^{\cdot }$$ 2

The heterogeneous Fenton process typically employed as catalyst materials such as magnetite, modified magnetite, and ferrites.^20,21^ Modified magnetite and ferrites are iron-based materials that incorporate different transition metals (TM) into the TM_*x*_Fe_3–*x*_O_4_ crystalline structure.^22,23^ This modification can enhance the efficiency of the Fenton process through a Fenton-like mechanism, where the inserted TM participates in the oxidation and reduction reactions involved in the hydroxyl radical (HO^·^) generation.^24^ Our research group has been focused on synthesizing ferrites from discarded batteries,^25^ which have already been applied to the degradation of municipal solid waste landfill leachate.^26^ These ferrites were selected for use in this study due to their magnetic properties, ease of synthesis, and stability. However, it is still necessary to better understand the changes to the surface of the catalyst after its application and its use under simulated real conditions, an aspect that was explored in this work following its use in a photoreactor.

In this context, the use of sunlight as a UV source significantly reduces the process costs, especially in tropical countries like Brazil, which experience high solar radiation throughout the year.^27,28^ Several studies aim to develop reactors for the application of photocatalytic processes.^29,30^ The compound parabolic concentrator (CPC) reactors are the most used solar reactors due to their numerous advantages. These include the use of direct and diffuse radiation from the solar spectrum, the absence of mechanical systems to track the sun, the simplicity, low cost, easy installation, operation, and maintenance.^31,32^

The CPC solar collectors can concentrate the sunlight and are often used with single- or dual-axis solar tracking systems.^30^ This ensures that the aperture area is always perpendicular to the direct solar radiation. The CPC consists of a reflecting surface that concentrates radiation on a tubular receiver located at the linear focus of the parabola.^33^

In this study, a continuous-flow, recirculating, lab-made solar CPC photoreactor has been designed and applied in a heterogeneous photo-Fenton process, utilizing solar radiation and ferrite from discarded batteries as the catalyst. The photoreactor has been constructed using recyclable and low-cost materials, and when combined with waste-obtained ferrite, it offers a novel approach for local-scale wastewater treatment, highlighting the innovation of this work. The methylene blue (MB) was used as the target compound to evaluate the efficiency of the application process. In addition, changes to the catalyst surface have been investigated after its use in order to assess its reusability and to understand the role of different metals incorporated into the ferrite structure on its activity in the Fenton reaction.

## Results and Discussion

### CPC Photoreactor Construction

The solar CPC photoreactor was constructed on a laboratory scale for application of the heterogeneous photo-Fenton process (Figure 1). It consists of four tubular reactors connected in series, capable of treating 4 L of effluents within a 2 h period. The glass reactors were connected using transparent rubber hoses, and an aquarium pump was utilized to circulate the effluent inside the system. Figure 1a shows the effluent circulating within the reactor at the start of the photo-Fenton process application. The fixed ferrites can be observed at the center of the tubes (Figure 1b). The neodymium magnets were positioned behind the structure. The figure also shows the decrease of the MB color after 1 h of irradiation. Figure 1c shows the reflective surfaces of the CPC reactor, having been constructed using cardboard plates coated with aluminum foil, aiming to utilize recyclable and accessible materials.

**Figure 1:**
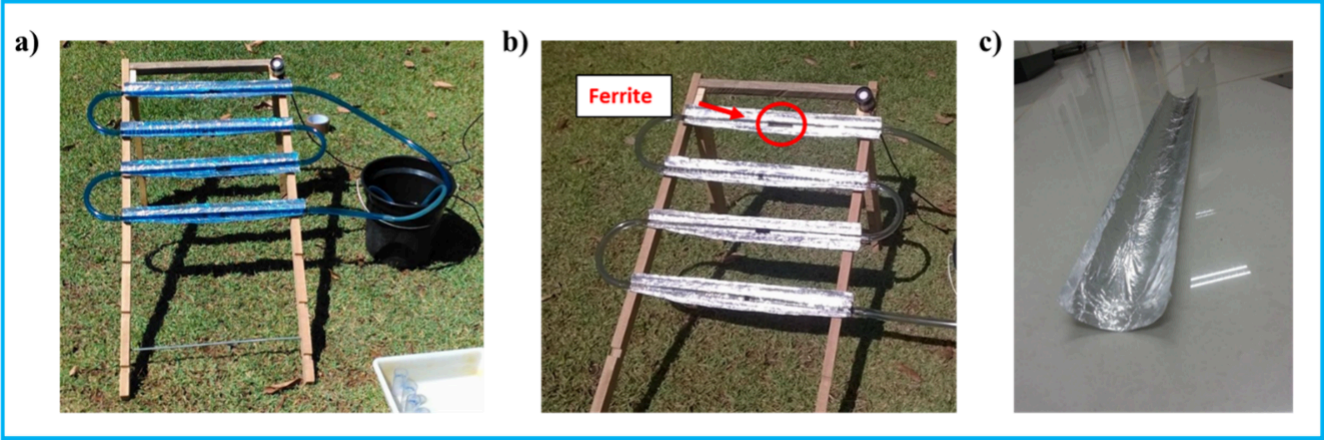
CPC reactor structure developed for heterogeneous photo-Fenton process application (a) at the beginning of the process; (b) after 1 h, with an indication of the catalyst fixation during application; (c) collecting surface.

The pipes and reflective surface dimensions are presented in Table 1. The kinematic viscosity of the initial effluents was thought to be equal to that of water for the calculations. The operating temperature of the reactor was set at 313 K, corresponding to the temperature at which the kinematic viscosity of water is 0.695 × 10^–6^ m^2^ s^–1^.^34^

**Table 1:** Dimensions of Pipes and the Reflective Surface

pipes		reflective surface	
nominal diameter	12 mm	concentration factor (CF)	1
internal diameter	10 mm	reflector surface width (D)	62.8 mm
length	520 mm	reflector surface height (H)	25.7 mm

The energy required to promote the fluid circulation considering the course and any irregularities was calculated based on the Bernoulli Equation (eq 3). In this equation, *P* represents the pressure exerted on the fluid, γ is the specific weight of the fluid, *g* is the gravitational constant, *v* denotes the velocity of the fluid, *z* represents the height, and *h*_T_ corresponds to the pressure drops (head loss) due to irregularities. The head loss can also be expressed through an equation (eq 4), where *L* denotes the actual length of the tube, *L*_e_ is the length considering all irregularities, *k* is a constant that is dependent on the type of irregularity, and *F* represents the friction factor. The friction factor is a dimensionless number and is determined by the Reynolds number (*Re*) and the ratio between the roughness (ε) and the diameter (*D*) of the tube (eq 5).^34^

$$\frac{P_{1}}{\gamma }+\frac{v_{1}^{2}}{2g}+z_{1}=\frac{P_{2}}{\gamma }+\frac{v_{2}^{2}}{2g}+z_{2}+h_{Τ}$$ 3

$$h_{Τ}=F.\frac{v^{2}}{2g}.\left(\frac{L}{D}+\sum_{i}\frac{L_{e}}{D}\right)+\sum_{i}k.\frac{v^{2}}{2g}$$ 4

$$F={\left[2\log\left(\frac{0.27\varepsilon }{D}+\left(\frac{7}{Re^{0.9}}\right)\right)\right]}^{-2}$$ 5

After the calculations, it was determined that a submersible aquarium pump would be sufficient to provide the necessary continuous flow. The fluid velocity was assessed in terms of the volume flow and found to be equal to 0.375 m^3^ h^–1^. The wooden support structure where the tubes were placed (Figure 1) was constructed with its orientation toward the Equator (north in the southern hemisphere) and inclined based on the geographic location in order to maximize solar capture. The inclination was determined to be 22°24′46″, according to the Meteorological Bulletin of Atmospheric Sciences from the Federal University of Itajubá.

Another crucial parameter for reactor application is the opening and height of the parabola to be shaped with the reflective surface (Figure 1c). These parameters were calculated considering the concentration factor (eq 6). The typical values for the reception half angle (θA) in photochemical applications range from 60 to 90°. This broad range allows the receiver to capture both direct and diffuse radiation, with the advantage of accommodating deviations from both the reflecting surface and the alignment of the receiving tube. This is significant in reducing photoreactor costs. To obtain a CPC with a cylindrical absorber tube, the “limit radius” principle was extended, considering the circular cross-section. It was determined that the maximum semiangle should be tangent to the absorption circle.^37^ Therefore, a reception semiangle of 90° was adopted, as for photocatalytic applications, achieving a concentration factor (CF) of 1. This theoretically allows for the capture of all direct and diffuse radiation. Additionally, the height of the parabola (*H*) and the width of the reflector surface (*D*) were calculated (eqs 7 and 8), with the results presented in Table 1 being for the developed reactor. With these parameters analyzed, sufficient information was obtained regarding the functioning of the CPC reactor. After development, the reactor was used to study the degradation of MB as a model compound to understand the developed photoreactor efficiency.

$$\mathrm{CF}=\frac{1}{\mathrm{sen}\;\theta_{A}}=\frac{D}{\pi D_{i}}$$ 6

$$H=\frac{\pi D_{i}}{2}\left[\frac{1}{\sin\;\theta_{A}\tan\;\theta_{A}}+\frac{1}{2}+\frac{1}{\pi \;\sin\;\theta_{A}}\right]$$ 7

$$D=FC\pi D_{i}$$ 8

### General Aspects of the Catalyst Used

The catalysts for the present study were Mn-Zn ferrites (Mn_*x*_Zn_1–*x*_Fe_2_O_4_), which were obtained by the citrate precursor method, and their synthesis has been previously described.^23^ As an ecofriendly alternative, spent batteries were used, as reported by previous work from our research group.^25^

Briefly, the crystalline Mn-Zn ferrites obtained had a cubic spinel structure with saturation magnetization of 37.04 emu g^–1^ and spherical morphology with heavy agglomeration due to particles’ magnetic characteristic (average particle size ranged from 80 to 150 nm).^23,25^ The material obtained has a good magnetization, which makes the catalyst suitable for the heterogeneous Fenton process application due to its easier separation after the process. The catalyst’s activity for real wastewater treatment was previously investigated,^26^ but only through batch experiments. In the present work, the use of a catalyst derived from spent batteries was studied as well as its immobilization in a constructed low-cost CPC photoreactor, its efficiency, and the chemical composition changes after application.

### Photo-Fenton Experiments Using the Constructed CPC Photoreactor

MB was selected as the target compound to assess the applicability of the developed CPC reactor and to optimize the conditions for the heterogeneous photo-Fenton process. Due to the use of solar radiation and an aquarium pump that does not allow precise control of the flow (although it was constant), parameters such as flow rate and system temperature were considered as variables. The studied and optimized parameters were the initial pH value of the solution, the hydrogen peroxide concentration, the radiation exposure time, and the amount of catalyst necessary in trying to achieve the best conditions for MB degradation.

The experiments were conducted with a MB solution of 20 mg L^–1^ without pH adjustment, which consisted of a pH value close to neutrality. The concentrations of the oxidizing agent and the catalyst were 10 mmol L^–1^ of H_2_O_2_ and 0.1% (w/v) ferrite (obtained from spent batteries), respectively. In the experiments mentioned, the average removal results obtained after 2 h of treatment showed that the accumulated energy was 30.622 J cm^–2^, resulting in the removal of 18% of MB, 30% of COD, and 7% of TOC. An apparent color removal has not been observed. After 30 min of the process of irradiation, all the H_2_O_2_ was consumed and replaced without significant degradation. This observed effect indicated a lower catalyst activity toward H_2_O_2_ decomposition at neutral pH. A study by Roonasi and Nezhad (2016),^35^ which compared the activity of Zn, Mn, Fe, and Cu ferrites in phenol degradation under acidic, neutral, and basic conditions, found that only Cu ferrite maintained its activity at neutral pH. This highlights the role of pH in influencing pollutant adsorption onto ferrite surfaces, which is governed by the surface charge of the catalyst and the molecular charge of the target pollutant. In addition, the heterogeneous Fenton degradation of phenol using CuFe_2_O_4_ showed a faster degradation at pH 2.6 than when compared to pH 4.4, 6.4, 8.4, and 10, while at pH 10, almost no phenol removal was observed.^36^

The investigations using different H_2_O_2_ concentrations (2–10 mmol L^–1^) and also catalyst concentrations (0.01–0.2% (w/v)) did not result in significant improvement to the MB degradation. The pH value was adjusted to 2.5, and the process was applied under the best conditions observed for MB degradation without pH adjustment, which were 10 mmol L^–1^ of H_2_O_2_ and 0.1% (w/v) of ferrite (Figure 2). The MB degradation reached values below the detection limit after 90 min of process application (Figure 2a), with a degradation kinetic constant equal to 0.04 min^–1^ (*R* ^2^ = 0.92) fitted to a pseudo-first-order model. Furthermore, after 60 min, it was possible to observe a visual discoloration (Figure 2b). In this experiment, a volume of 4 L of the initial MB solution was treated during the period of highest solar irradiance (11 a.m.–1 p.m.).

**Figure 2:**
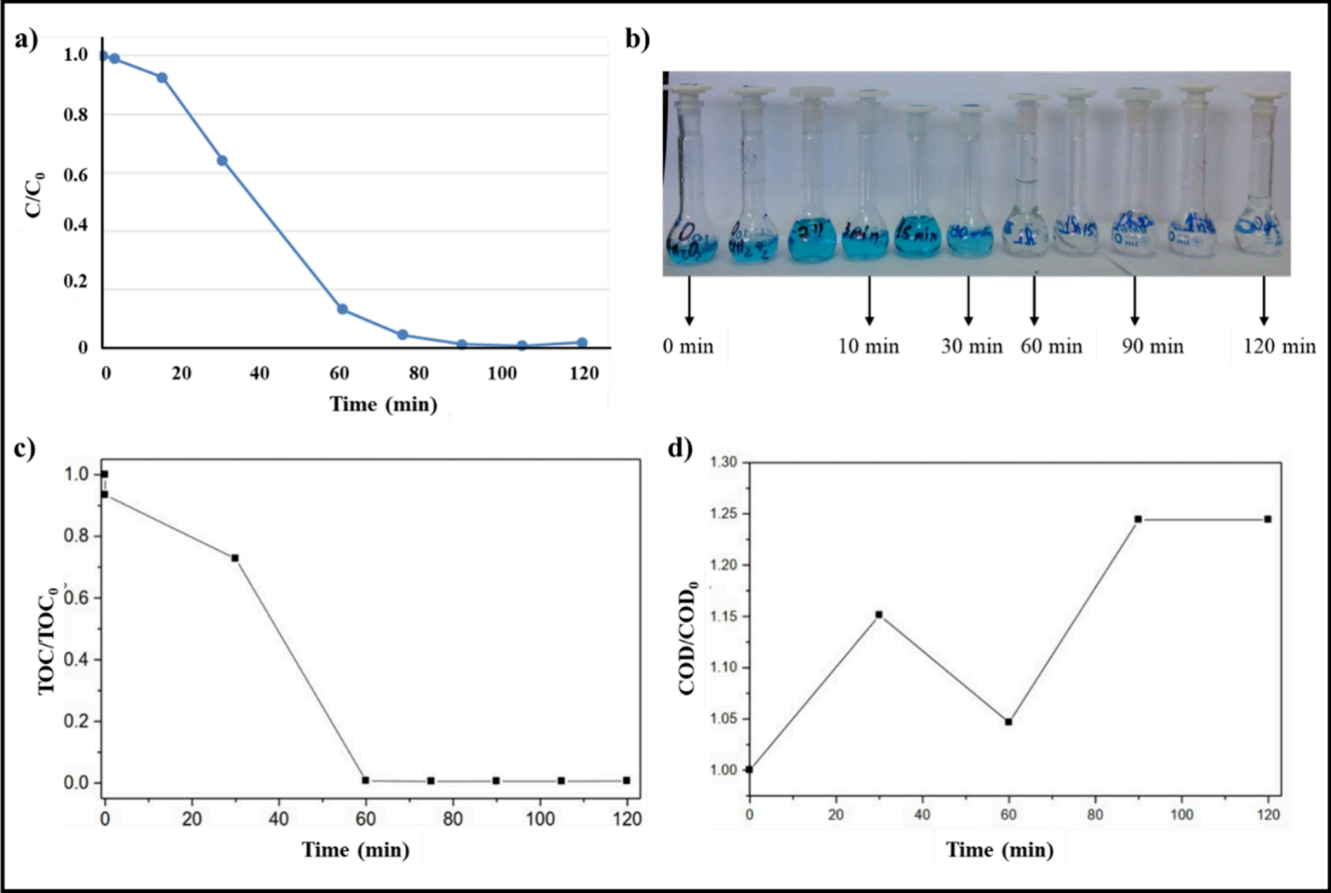
(a) MB degradation, (b) color removal, (c) TOC removal, and (d) COD variation during heterogeneous solar photo-Fenton process application. Ferrite = 0.1% (w/v); [MB] = 20 mg L^–1^; [H_2_O_2_] = 10 mmol L^–1^, pH = 2.5.

Controlled experiments were also studied, and the results indicated low photocatalytic activity of ferrites for MB degradation. Additionally, ferrites also showed a low dye adsorption capacity. Experiments using only solar radiation demonstrated a lower contribution for direct photolysis compared to the Fenton process. The UV and hydrogen peroxide combination also showed a dye degradation but with lower removal compared to the application using the heterogeneous photo-Fenton process. This result suggests that ferrites exhibit good catalytic activity in the heterogeneous photo-Fenton process for MB degradation. The developed ferrites have already been applied to the degradation of leachates from municipal solid waste landfills, displaying high catalytic activity during the implementation of the heterogeneous photo-Fenton process following a physical-chemical process.^26^

Other materials with similar structures have also been successfully applied to contaminant degradation. Guardiano et al.^37^ proposed a copper-modified magnetite for azithromycin, clarithromycin, and sulfamethoxazole degradation, applying the heterogeneous photo-Fenton process, and observed almost 50% of those antibiotics removal after 120 min using 15 mmol L^–1^ of H_2_O_2_ and 0.125 g L^–1^ of the catalyst at pH 8, even in complex matrices such as wastewater treatment plant (WWTP) effluents. Nguyen et al.^38^ studied the effect of different salt precursors on Mn-Zn ferrite obtention and its effect on rhodamine-B (RhB) degradation when applying the Fenton process. The authors observed more than 70% of RhB removal after 60 min of process application with the use of 1 g L^–1^ of the ferrites, 10 mmol L^–1^ of H_2_O_2_ and adjusting the solution pH to 4.^38^ However, these studies were done using UV-C radiation or visible light in batch experiments (maximum volume of 300 mL), which is different from the solar light and 4 L of MB solution used in this work.

The discoloration of the MB solution occurred gradually (Figure 2b). However, discoloration alone is insufficient to assess MB degradation due to the possibility of byproduct formation that does not absorb in the visible wavelength region. Therefore, total organic carbon (TOC) analyses were conducted in order to examine its variation during the study of the heterogeneous photo-Fenton process (Figure 2c). Furthermore, the possible change in the chemical oxygen demand (COD) (Figure 2d) was also investigated. The COD values provide information on the amount of dissolved oxygen consumed in the medium, and that can be used as an indirect parameter to evaluate the degradation of organic compounds.

After 60 min of irradiation, 98% of the TOC was removed (Figure 2c), indicating the mineralization of the degradation of MB and the possible byproducts generated during the process. There is also the possibility that some products were adsorbed onto the catalyst, and the removal of TOC occurs through a combination of adsorption and oxidation processes. The study monitoring the COD values showed an increase in this parameter during the heterogeneous photo-Fenton process (Figure 2d). One possible explanation is that the COD analysis is based on a colorimetric method, which is susceptible to errors, such as the generation of byproducts and the presence of inorganic ions in the solution, which can interfere with the analysis.^39^

After 30 min of irradiation, the H_2_O_2_ concentration had decreased to 0.011 mmol L^–1^, and at this point, an additional increment of peroxide was made. After that, the MB degradation continued (Figure 2a) until total mineralization of the MB or possible generated byproducts (Figure 2c). The H_2_O_2_ concentration measured after 120 min of the photo-Fenton process was 0.013 mmol L^–1^.

Different authors studied the heterogeneous photo-Fenton process application to MB degradation.^40,41^ Wang et al. observed 100% of MB color removal after 60 min of process application at pH 3 using Fe­(II)­Fe­(III)-LDH as the catalyst,^41^ which is similar to the results obtained by this work. Nevertheless, it should be emphasized that the MB removal achieved in the present work was obtained using a continuous flow photoreactor and a total treated volume of 4 L, which presents greater challenges compared with batch experiments.

The proposed reactor, although applied to the photo-Fenton process, may be used for the degradation of other contaminants through different photocatalytic processes.^29^ Different catalysts can also be employed; however, the effect of catalyst dispersion or immobilization in the reactor must be evaluated. The reactor is also suitable for homogeneous process applications and treatment of effluents produced on a laboratory scale. Some studies have focused on the development of CPC reactors.^30,33^ Since the proposed solar photoreactor was constructed using low-cost and recyclable materials, it is easy to assemble and suitable for developing countries with high solar incidence.^27^

The CPC reactor plays a crucial role in enhancing the efficiency of the photo-Fenton process application. By directing the radiation source straight to the tube where the photocatalytic process occurs, the CPC reactor increases the effectiveness of the photo-Fenton process, particularly in homogeneous media. In countries with abundant sunlight, the CPC reactor offers an excellent means to harness the abundance of solar energy for contaminant degradation. The catalyst used in the developed system exhibits low activity under neutral and alkaline pH conditions. However, it is important to highlight that the photoreactor itself is versatile and can operate across a wide pH range or be adapted for different AOPs (or even process combinations) by modifying the catalyst or adjusting the process and operational parameters.

The engineering involved in constructing the homemade solar CPC photoreactor, as previously discussed, has contributed to the development of a reactor that closely approximates ideal conditions for treating a 4 L volume of effluent. The optimal parabolic curve was calculated to determine the best curvature for achieving a CF (concentration factor) of 1. All of the calculations performed for the assembly have facilitated a faster phase of obtaining results in the experimental tests conducted using the reactor. The optimal experimental conditions for the application of the process and MB degradation using the heterogeneous solar photo-Fenton process are presented in Table 2. Additionally, the required use of 10 mmol L^–1^ of H_2_O_2_, which was fully consumed after 30 min, may contribute to increased operational costs. However, the overall efficiency of the proposed system, combined with the use of low-cost and recyclable materials, the synthesis of the catalyst from spent batteries, and the utilization of solar light as the activation source, makes this approach a low-cost and economically attractive alternative compared to other systems reported.

**Table 2:** Optimized Experimental Conditions for a Homemade Solar CPC Photoreactor Applied to MB Degradation by the Heterogeneous Solar Photo-Fenton Process

item	description
photoreactor	solar CPC
process	photo-Fenton
exposed area	0.065312 m^2^
pump	Sarlobetter S300
flow	0.127 m^3^ h^–1^
total irradiated volume	0.004 m^3^
number of tubes	4
catalyst	ferrites from spent batteries
initial pH	2.5
exposure time	2 h
schedule of process application	11 a.m.–13 p.m.
fluid velocities	*V*_glass_ = 0.45 m s^–1^
	*V*_PVC_ = 0.31 m s^–1^
Reynolds	*Re*_glass_ = 6462.89
	*Re*_PVC_ = 5385.74
volume of 1 tube	0.04 L
initial conditions	MB initial concentration: 20 mg L^–1^
	temperature: 20–30 °C
	pressure: 756 mmHg ∼ 1 atm
	ferrite concentration: 0.1% (w/v)
catalyst position	magnet supported ferrite

### Surface Modification after Process Application

A catalyst must fulfill certain requirements to qualify for wastewater treatment applications.^42–44^ Among these, it is important to evaluate the catalyst leaching, after the process application, and its ability to sustain catalytic activity over multiple cycles or extended periods of use without significant loss of efficiency.^45^ Therefore, it is important to identify the bonding states of the elements present in the catalyst before and after the process application,^46^ as the preservation of these chemical states plays a pivotal role in maintaining its catalytic performance.

In this scenario, atomic absorption spectroscopy (AAS) analysis and X-ray photoelectron spectroscopy (XPS) characterization were performed before and after the catalyst had been used in the constructed CPC photoreactor (Figure 3). Before the application of the process, no Fe, Mn, or Zn were detected in the solution. However, after the process, the concentrations of these metals were found to be 1.629; 9.767 and 2.549 μg L^–1^, respectively, indicating a relatively low leaching effect. The survey scan obtained before the process application (Figure S1) showed the presence of some impurities in the sample beyond the Mn, Zn, Fe, O elements, such as K, Na, and Cl. These impurities were observed as a consequence of the spent batteries’ composition.^25^

**Figure 3:**
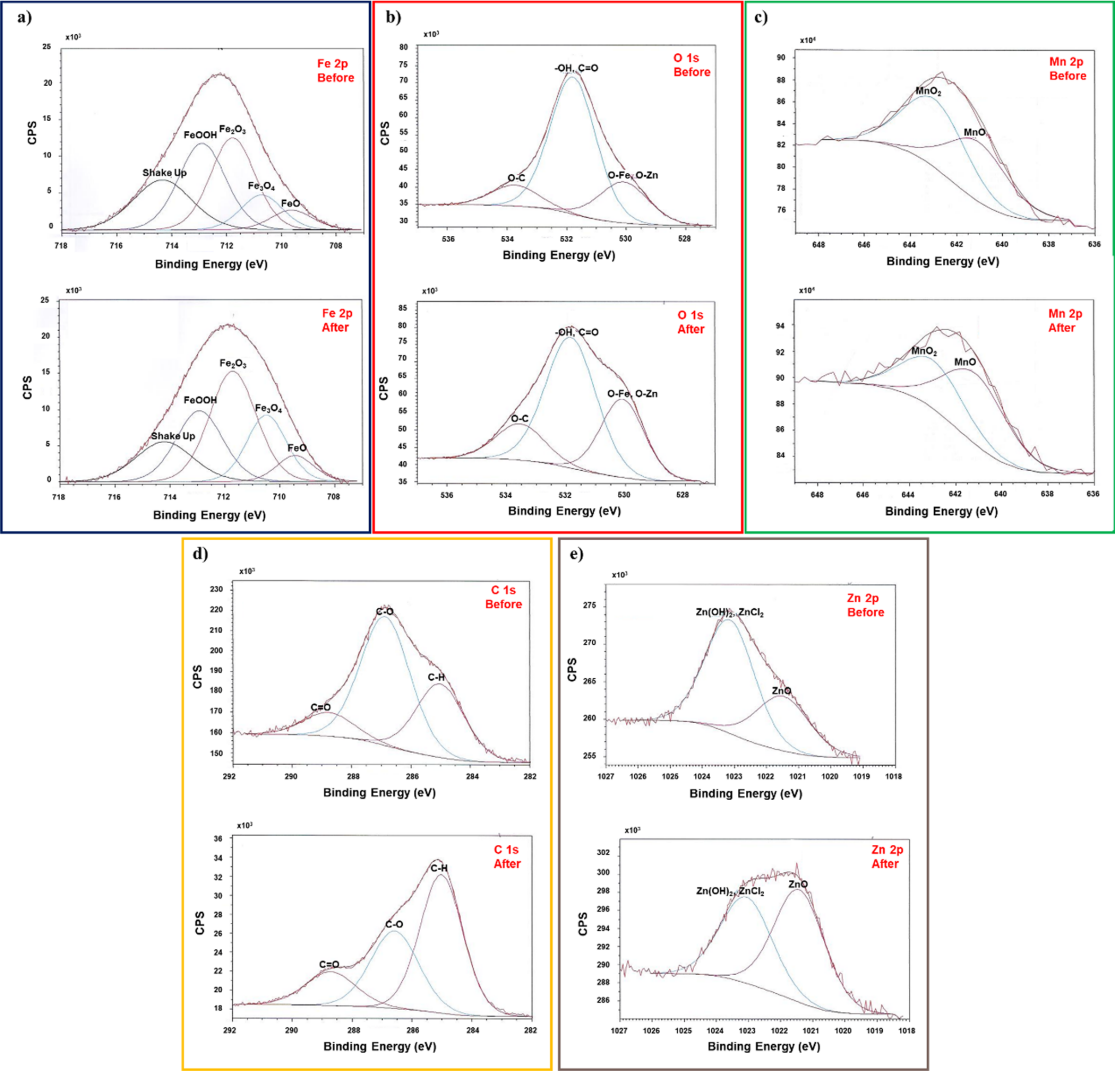
High-resolution XPS spectra of (a) Fe 2p, (b) O 1s, (c) Mn 2p, (d) C 1s, and (e) Zn 2p before and after the catalyst’s use in the heterogeneous photo-Fenton process carried out in the developed lab-made CPC photoreactor.

The Fe 2p_3/2_ high-resolution spectra (Figure 3a) were deconvoluted into five components (709.5, 710.3, 711.7, 712.9, and 714.5 eV). These components were associated, respectively, to Fe^2+^, Fe_3_O_4_, Fe^3+^ (Fe_2_O_3_ and FeOOH), and Fe^3+^ shakeup satellite.^46^ Before the process, a predominance of the Fe^3+^ species (first iron surface monolayer) can be observed. After the process application, the Fe 2p_3/2_ high-resolution spectra slightly dislocate to lower energy, and a partial Fe^3+^ reduction with an increase in the Fe_3_O_4_ species can be observed. This reduction suggests that the catalyst still maintains its active phase during the Fenton process and can be reused, being associated with the photo-Fenton catalytic reduction of Fe^3+^ to Fe^2+^.^47^ In addition, the sample was magnetic, which also reinforces the Fe_3_O_4_ presence.^48^

The O 1s high-resolution spectra (Figure 3b) can be deconvoluted into three components. The first peak at 533.5 eV is associated to C–O bonds, while the second peak corresponds to O–H groups (oxygen vacancies^45^) and C=O bonds. The third peak at 529.8 eV can be attributed to the O–Fe, O–Zn, and O–Mn bonds.^49^ A stronger presence of hydroxyl groups and surface contamination was observed on the catalyst before the process application, likely due to its obtention from spent batteries. After the process, a decrease in the intensity of the O 1s signal has been observed, particularly in the hydroxyl bonds, due to C contamination on the surface, as highlighted by the C 1s spectra (Figure 3d) before and after the treatment. This change can be associated to a slightly reduced, yet still significant, adsorption activity of ferrites, as reported by different authors.^50,51^

The Mn 2p high-resolution spectra after this process (Figure 3c) can be deconvoluted into two components (MnO_2_ and MnO). Following the process application, a reduction of MnO_2_ species and the formation of a greater amount of MnO (Mn^2+^) have been observed. A similar trend was observed for Zn in the Zn 2p spectra (Figure 3e), with an increase in the number of ZnO species. These results suggest that the Mn element, when inserted into the ferrite structure, is involved in the catalytic mechanisms of the Fenton reaction, undergoing oxidation and reduction to generate hydroxyl radicals (eq 9).^52^ In addition, the Zn presence can act by modifying the material band gap and decreasing recombination, as observed for a different author that studied the ZnFe_2_O_4_ activity in the Fenton process.^53^

$${\mathrm{Mn}}^{2+}+H_{2}O_{2}\to {\mathrm{Mn}}^{3+}+{\mathrm{HO}}^{\cdot }+{\mathrm{HO}}^{-}$$ 9

Finally, a schematic mechanism for the use of ferrites obtained from spent batteries in the CPC photoreactor can be proposed (Figure 4). Due to the catalyst’s low photocatalytic activity and low adsorption capacity, the main degradation pathway of MB relies on the generation of reactive oxygen species, such as HO^·^, on the Mn–Zn ferrite surface, with contributions from Mn, Zn, and Fe through their redox processes. After being used in the CPC photoreactor, the catalyst was reduced and maintained its active phase, making it suitable for further catalytic cycles. The incomplete mineralization of MB within 60 min of process application required the recirculation of the effluent (containing MB and its transformation products) through the constructed reactor. However, after 60 min, the process showed to be efficient for MB degradation.

**Figure 4:**
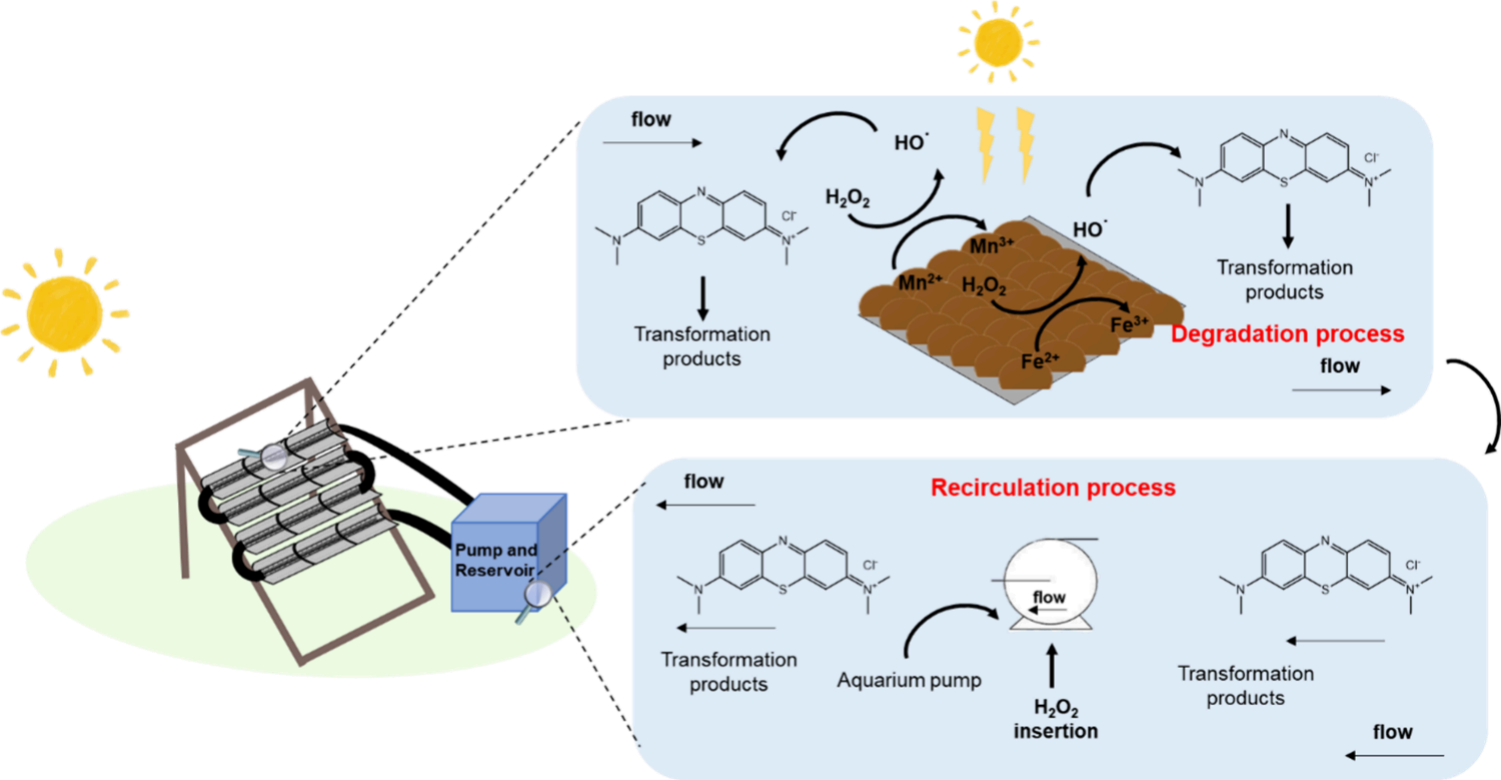
Proposed mechanism of Mn-Zn ferrites obtained from spent batteries is incorporated into the CPC photoreactor for efficient MB degradation.

In the present work, MB was chosen as the target compound due to its availability and the existing knowledge reported in the literature regarding the mechanisms involved in its degradation.^54^ There have also been studies on the generation of byproducts after applying the photo-Fenton process using a small volume of MB in a batch bench.^55^ Although the developed CPC reactor was designed for this study, it enables future expansion studies, including the degradation of emerging contaminants, the evaluation of new photocatalytic materials using solar irradiation, and the investigation of real effluent treatments, such as those generated at our university.

## Final Remarks

The application of the lab-made reactor for the heterogeneous photo-Fenton process yielded high efficiency in MB removal, with a 98% mineralization of the dye solution being achieved after 1 h of solar irradiation. This project demonstrates that even with simple and recyclable materials, it is possible to construct a CPC reactor that can be used in heterogeneous solar photo-Fenton reactions. In this case, ferrite, obtained from spent batteries, was employed as the catalyst, which maintained its performance even after use in the reactor. In addition, the developed lab-made solar CPC photoreactor can be used for investigating various photocatalytic processes and exploring new materials in research studies.

## Materials and Methods

### Chemicals and Catalyst Synthesis

MB (Synth), hydrogen peroxide 32–36.5% (w/w) (Synth), NaOH (Synth), HNO_3_ (Vetec), and H_2_SO_4_ 95–98% (Kollins) were used in this work. The catalyst synthesis and characterization were published in a previous work.^25,26^ Herein, in this work, we studied the catalyst surface modification before and after the photo-Fenton process using XPS analysis. A UNI-SPECS UHV system equipped with an Mg Kα X-ray source (hν = 1253.6 eV) at a pressure of <5 × 10^–7^ Pa was used. The inelastic backgrounds of the Fe 2p3/2, Zn 2p, Mn 2p, C 1s, and O 1s spectra were subtracted using Shirley’s method. The surface layer composition was determined based on the relative peak areas, corrected by the Scofield sensitivity factors of the corresponding elements, and the binding energy scale was corrected using the C 1s hydrocarbon group (284.8 eV). The spectra were deconvoluted using a Voigt function with a combination of Gaussian (70%) and Lorentzian (30%) components. The full width at half-maximum (fwhm) varied between 1.6 and 2.1 eV, and the precision in the composition determination was within ±10%. The peak positions were determined with a precision of ± 0.1 eV.

### CPC Reactor Design and Optimization

The reactor was supported by a wooden structure inclined at 22.41°, following the latitude of Itajubá, MG - Brazil. Borosilicate glass tubes, obtained from discarded burets from chemistry laboratories, were used as reactors and connected with transparent rubber tubes. A plastic container was used to store the effluent to be treated and to adjust the pH value if necessary. Additionally, this container served as a storage source for the MB solution, facilitating recirculation within the system. To propel the fluid through the tubes, a submersible aquarium pump (Sarlobetter S300) was employed. A radiometer (PMA2100 SOLAR LIGHT) was installed on the reactor support to measure the intensity and summation of the solar UV rays during the experiment.

### Degradation Experiments

A standard aqueous solution of 1.0 g L^–1^ of MB dye was prepared and used for all of the solutions in the degradation tests, as well as for the calibration curve. The photodegradation experiments were conducted using 0.1% ferrite, calculated in relation to the reactor volume (w/v), and 4 L of the MB dye solution (20 mg L^–1^). H_2_SO_4_ was gradually added to the MB solution to reach a pH of 2.5. The aquarium pump was then immersed in the solution, and 10 mmol L^–1^ hydrogen peroxide was immediately added to initiate the reaction. The photodegradation experiments in the CPC reactor were carried out at the Federal University of Itajubá, located at Minas Gerais state, Brazil (Coordinates: 22°24′ S 45°26′ W), during the winter period. Since the composition of solar radiation can vary daily, a radiometer was used to measure the radiation levels. Samples were collected at predetermined time intervals or at the same energy dose. Subsequently, the solution was left undisturbed until the hydrogen peroxide was completely consumed.

### Chemical Analyses

The TOC determination has been performed by using an Analytik Jena Multi N/C 2100S instrument to monitor the MB mineralization during the experiments. Prior to each determination, the samples were filtered using 0.45 μm membranes (*Química Moderna*). The residual hydrogen peroxide was measured using the metavanadate method throughout the experiments.^56^ The MB degradation was monitored by measuring the absorbance at 660 nm using a METERSPECTRUM2000 spectrophotometer. Due to the high absorption of the MB solution, the collected samples were diluted five times before the measurements. The COD determination has been performed using the colorimetric method.^57^ AAS analysis was used to measure ion leaching after the process application.

## Supplementary Material



## Notes

XPS survey scan graphs (PDF)
