# Supplementary material for: Continuous-Flow Solar Heterogeneous Photo-Fenton Process in a Lab-Made Low-Cost CPC Photoreactor for Efficient Dye Degradation
Source: ACS Omega. 2025 Jul 15;10(29):32216–25. doi: 10.1021/acsomega.5c03994 (PMC12311708; doi:10.1021/acsomega.5c03994)

Supporting Information

Continuous-Flow Solar Heterogeneous Photo-Fenton Process in a Lab-Made Low-Cost CPC Photoreactor for Efficient Dye Degradation

Matheus Gabriel Guardiano[1,*], Yara Silvestrine e Silva[1], Rossano Gimenes[1], Sandro José de Andrade[1], Márcia Matiko Kondo[1], Milady Renata Apolinário da Silva[1,*]

[1]Federal University of Itajubá, Av. BPS, 1303, Itajubá – MG, 37500-901, Brazil.

*Corresponding authors: milady@unifei.edu.br, mggds15@gmail.com

Fig. S1. XPS survey scan **a)** before and **b)** after the process application.

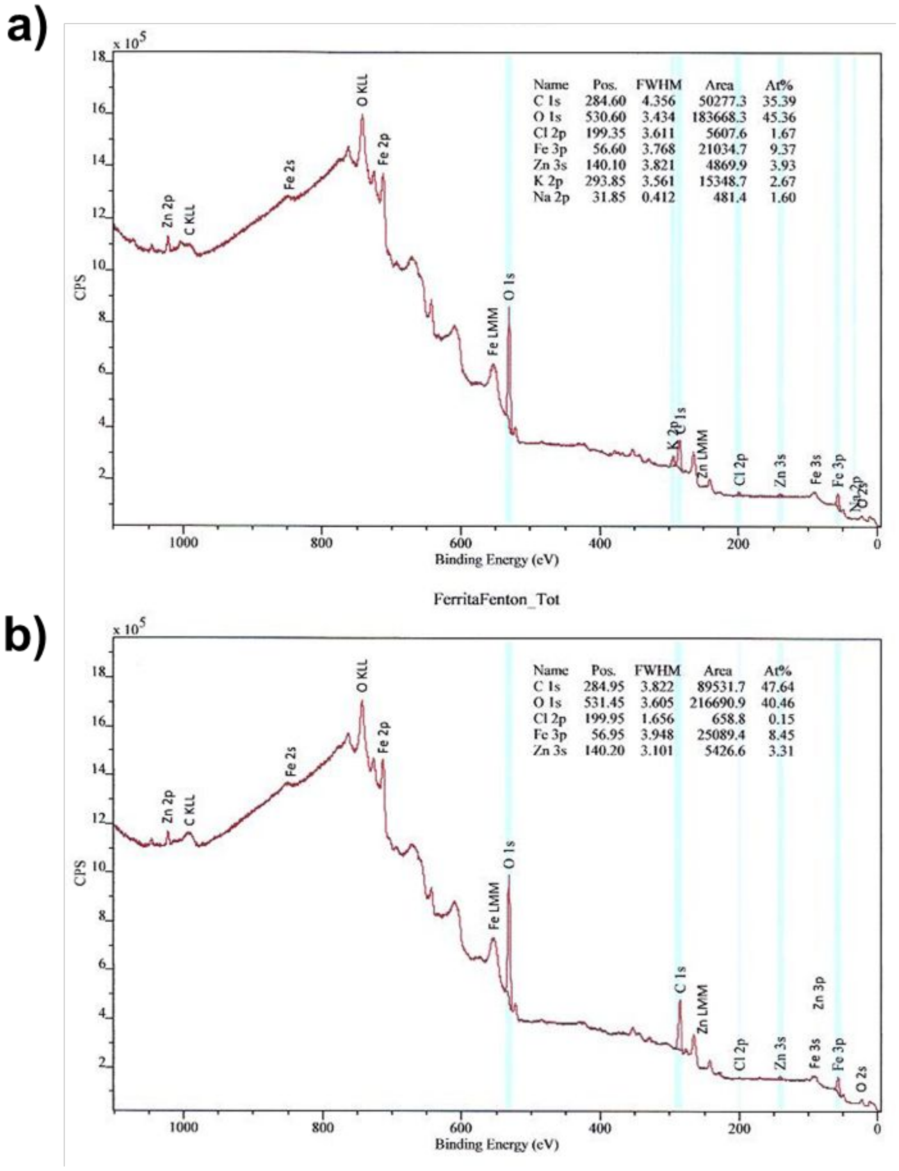

Supplement: Supplementary file 1 [file ao5c03994_si_001.pdf]
